# Integrated network analysis identifying potential novel drug candidates and targets for Parkinson's disease

**DOI:** 10.1038/s41598-021-92701-2

**Published:** 2021-06-23

**Authors:** Pusheng Quan, Kai Wang, Shi Yan, Shirong Wen, Chengqun Wei, Xinyu Zhang, Jingwei Cao, Lifen Yao

**Affiliations:** 1 Department of Neurology, The First Affiliated Hospital, Harbin Medical University, Harbin, 150081 China; 2 Center of TOF-PET/CT/MR, The Fourth Affiliated Hospital, Harbin Medical University, Harbin, 150081 China; 3 Department of General Practice, Heilongjiang Provincial Hospital, Harbin, 150081 China

**Keywords:** Neurology, Neurological disorders, Data mining

## Abstract

This study aimed to identify potential novel drug candidates and targets for Parkinson’s disease. First, 970 genes that have been reported to be related to PD were collected from five databases, and functional enrichment analysis of these genes was conducted to investigate their potential mechanisms. Then, we collected drugs and related targets from DrugBank, narrowed the list by proximity scores and Inverted Gene Set Enrichment analysis of drug targets, and identified potential drug candidates for PD treatment. Finally, we compared the expression distribution of the candidate drug-target genes between the PD group and the control group in the public dataset with the largest sample size (GSE99039) in Gene Expression Omnibus. Ten drugs with an FDR < 0.1 and their corresponding targets were identified. Some target genes of the ten drugs significantly overlapped with PD-related genes or already known therapeutic targets for PD. Nine differentially expressed drug-target genes with p < 0.05 were screened. This work will facilitate further research into the possible efficacy of new drugs for PD and will provide valuable clues for drug design.

## Introduction

Parkinson's disease (PD) is a pervasive, progressive, disabling neurodegenerative disorder with motor and nonmotor features^1^. PD places a significant burden on society and the affected individuals, and approximately 6.1 million people worldwide had been diagnosed with PD in 2016^2^. Dopamine depletion leading to hyperactivity of the corticostriatal glutamatergic pathway is thought to be primarily responsible for parkinsonian symptoms such as resting tremors, rigidity, dyskinesia and postural instability^3,4^. Levodopa is the gold standard drug that provides symptomatic relief from motor problems but it has some side effects, and its effectiveness is reduced under long-term treatment. In the present context, medication (including dopamine agonists and monoamine oxidase B inhibitors) and invasive surgery (deep brain stimulation) are being used to reduce the shortcomings of levodopa therapy^5^. These therapies are quite helpful but are not always completely satisfactory. Thus, it is imperative to identify and develop promising drugs for preventing and treating PD.

Unfortunately, drug discovery is expensive and time-consuming. New drug development is affected by many factors, and 85.1% of potential drugs for PD tested thus far have failed in the clinical trial phase^6^. Under such a situation, the repositioning of available drugs for other disorders as potential novel therapeutic agents for PD becomes an ideal approach. A well-known example of drug repositioning against cancer is thalidomide, which was initially used as a sedative drug^7^. Network pharmacology is a commonly applied strategy that analyzes biological systems and establishes a drug-target-disease network for drug repositioning^8^. Many computational methods based on transcriptomic data have also been developed. Chuan et al. found 10 drugs that had certain therapeutical effect on PD based on a handful of genes^9^. Hindol et al. developed a bidirectional drug repositioning method to find out new drugs for PD^10^. However, most previous studies have mainly focused on certain specific genes and neglected the gene expression signatures. Here, we present an integrated method for the comparisons of gene expression signatures between a disease model and drug-treated condition network, prediction of drug-protein interactions, and large transcriptomic dataset mining.

The purpose of this scheme is to predict and identify new related drugs and targets by applying integrated network pharmacology and transcriptome analysis. Our work will facilitate further studies for better preventive strategies for PD.

## Results

### PD-related genes

**Figure 1:**
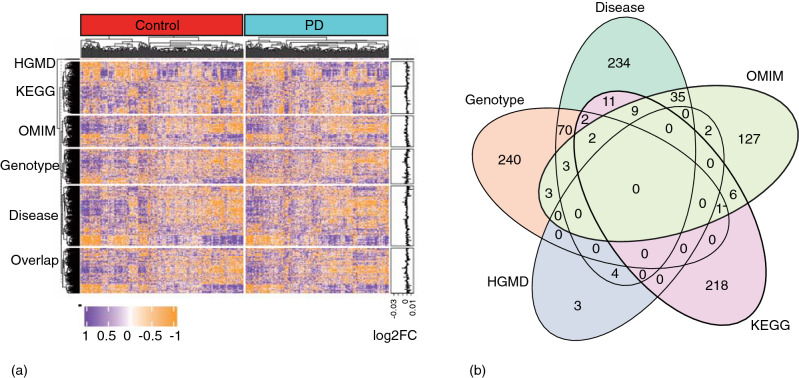
PD-related genes. **(a)** Heatmap for comparison between the control samples and patients with PD. Colors correspond to standardized log2-transformed expression values. Heatmap created using R and “pheatmap” package^48^. **(b)** Veen diagram show the number of PD-related genes collected from five databases. PD, parkinson’s disease. Veen diagram created using R and “ggVennDiagram” package^49^.

Genes from the five databases were integrated to include as many PD-related genes as possible. Specifically, 249 genes were retrieved from the Parkinson’s disease pathway (hsa05012) included in KEGG. By querying the keyword “Parkinson’s disease”, 188 genes were retrieved from OMIM, 9 genes from HGMD, 524 from Genotype, and 370 from DISEASE. After filtering, a total of 970 genes were retained as the PD-related genes. The expression levels of these genes in the PD and control subjects (GSE99039) are shown in the heatmap (Fig. 1a). Detailed provenance information for the genes is given in a Venn diagram (Fig. 1b).

### Protein–protein interactions between the PD-related genes

**Figure 2:**
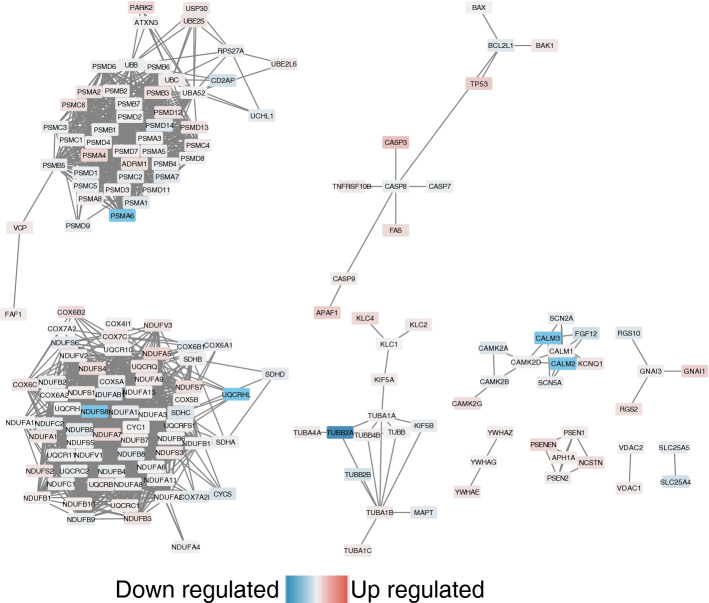
The constructed PPI network with PD-related genes. The red nodes represent up-regulated genes and the blue represent down-regulated genes in GSE99039. Visualization tool: Cytoscape (3.7.2). https://cytoscape.org/.

The PD-related genes were exported to the STRING, PINA, and HuRI databases to construct the PPI network. STRING contained 1723 interactions among 188 genes/proteins after removing all interactions with a combined score < 0.9; PINA contained 1127 interactions among 107 genes/proteins; and HuRI predicted 1411 experimental validation interactions of 119 genes/proteins (see Supplementary Fig. S1 online). We extracted 163 nonredundant genes/proteins and 1709 interactions by comparing the results from the three databases (Fig. 2). Nodes represent PD-related genes/proteins and edges represent interactions of these genes/proteins.

### Enrichment analysis of the PD-related genes

**Figure 3:**
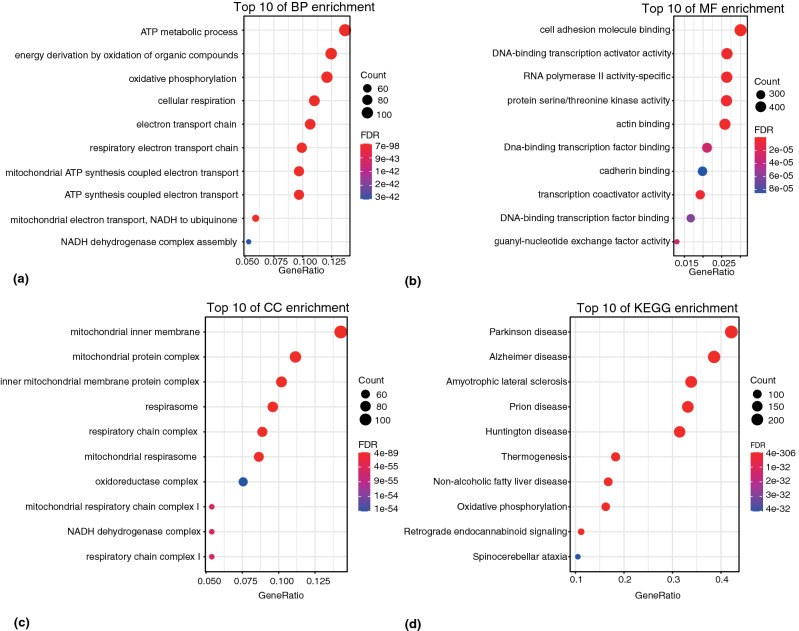
GO and KEGG enrichment analysis of the PD-related genes. **(a)** Biological process analysis. **(b)** Molecular function analysis. **(c)** Cellular component analysis. **(d)** KEGG pathway analysis. The color of the node represents significance, and the size of the node represents the number of genes enriched into the function.

In this study, based on GO and KEGG analysis of PD-related genes, several enriched biological processes and metabolic pathways were identified. The top 10 GO enrichment terms in the three GO categories and the KEGG pathways were illustrated by a bubble diagram. GO enrichment analysis of biological processes (BP) revealed that PD-related genes were mainly involved in the ATP metabolic process, energy derivation by oxidation of organic compounds, and oxidative phosphorylation (Fig. 3a). Molecular function (MF) analysis showed that these genes may take part in the cell adhesion molecule binding, DNA-binding transcription activator activity, and RNA polymerase II activity-specific processes (Fig. 3b). For cell component (CC) analysis, our genes mainly enriched in mitochondrial inner membrane, mitochondrial protein complex, and respirasome (Fig. 3c). KEGG pathway analysis indicated their involvement in Parkinson, Alzheimer, amyotrophic lateral sclerosis, prion disease, and Huntington disease (Fig. 3d).

### Network-based proximity between drugs and PD

**Figure 4:**
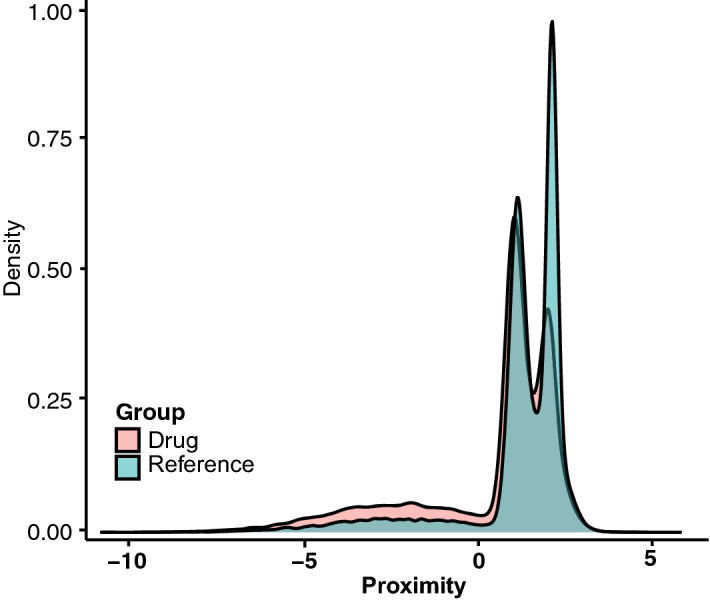
Proximity between drugs and PD. Density plot showing the distance distribution of all drugs to PD-related genes (pink) and the reference data (blue).

We tidied up the list of proximal drugs and excluded drugs irrelevant to PD by applying a network-based proximity analysis. The density plot showed that the distance distribution of drugs to PD-related genes overlapped but differed significantly from that of the reference data in the range of − 10.0 ~ 5.0 (Fig. 4). The overlap can be observed visually at the point of 1.0 ~ 2.0, suggesting that drugs in this range were unlikely to be treatment candidates for PD while drugs with distance < 1.0 might be effective for PD treatment. Thus, we took 1.0 as the threshold to screen candidate drugs for PD and to exclude any irrelevant drugs.

### Calculation of drug signatures

**Figure 5:**
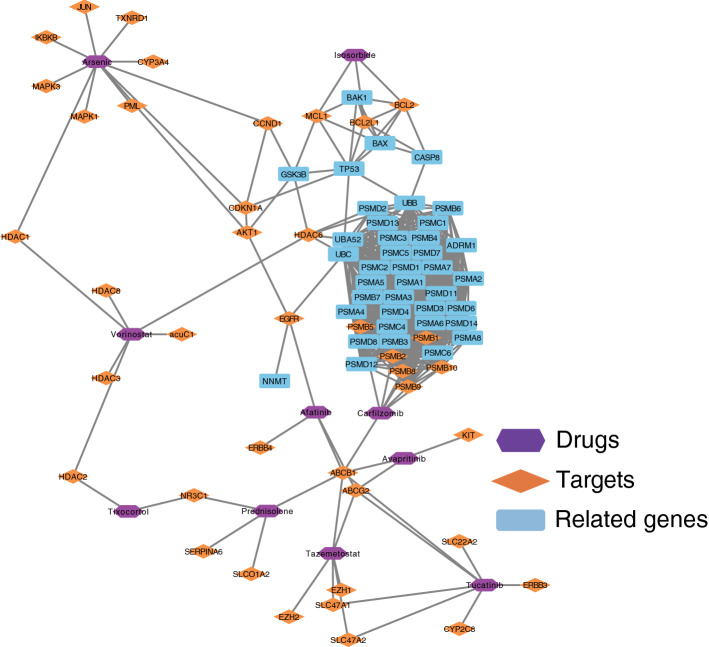
Building and analysis of drug-target-gene network. Purple nodes represent drug candidates, orange diamonds represent drug targets, and blue rectangles represent PD-related genes. Visualization tool: Cytoscape (3.7.2). https://cytoscape.org/.

Ultimately, 46 drugs with an FDR < 0.25 were identified. They may significantly influence PD-related genes. The drugs with FDR < 0.1 (ten drugs) and the corresponding targets (fifty-one targets) are shown in Table 1 and could potentially be options for therapies. Some target genes of the ten drugs significantly overlapped with the PD-related genes or the known therapeutic targets for PD. Additionally, we explored and visualized the interactions between the ten predicted drugs, their corresponding targets, and the PD-related genes (Fig. 5).

**Table 1:** Prediction of drug target and distance information by top10 (FDR < 0.1).

Sig-drug	Proximity	FDR	Targets
Carfilzomib	− 4.769	0.00	ABCB1, PSMB5, PSMB1, PSMB2, PSMB8, PSMB9, PSMB10
Afatinib	− 4.737	0.02	EGFR, ABCB1, ABCG2, ERBB4
Prednisolone acetate	− 4.989	0.02	ABCB1, SLCO1A2, NR3C1, SERPINA6
Isosorbide	− 4.989	0.03	BCL2, BCL2L1, MCL1
Arsenic trioxide	− 4.557	0.04	CYP3A4, TXNRD1, AKT1, JUN, IKBKB, MAPK3, CCND1, MAPK1, CDKN1A, HDAC1, PML
Vorinostat	− 4.638	0.04	HDAC2, HDAC1, HDAC8, HDAC3, HDAC6, ACUC1
Tucatinib	− 4.516	0.05	ABCB1, ABCG2, SLC22A2, CYP2C8, SLC47A1, SLC47A2, ERBB3
Tazemetostat	− 4.656	0.05	ABCB1, ABCG2, SLC47A1, SLC47A2, EZH2, EZH1
Avapritinib	− 4.611	0.05	ABCB1, ABCG2, KIT
Tixocortol	− 4.989	0.09	HDAC2, NR3C1

### Differentially expressed drug-target genes analysis

**Figure 6:**
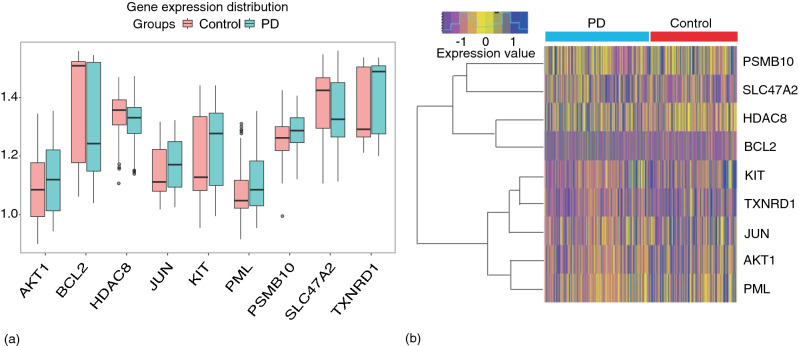
Differentially expressed drug-target genes analysis. (**a**) A box plot showing the differential expression distribution of nine drug-target genes between two groups. (**b**) Heatmap and clustering of nine differentially expressed drug-target genes.

We took the intersection of DEGs and drug-target genes, and nine differentially expressed drug-target genes with p < 0.05 were screened. Then, we compared the expression distribution of these genes in the two groups and visualized them with a box plot (Fig. 6a). In the final step, the heatmap and clustering tree revealed a distinct expression pattern of nine genes between the groups (Fig. 6b). PSMB10, SLC47A2, HDAC8, and BCL2 were clustered into the same model, while KIT, TXNRD1, JUN, AKT1, and PML were clustered into another model, suggesting that these nine targets may act through two distinct mechanisms. Further GO enrichment analysis of biological processes (BP) revealed that the nine genes were mainly involved in the response to oxidative stress. KEGG pathway analysis revealed that they were enriched in the apoptosis, neurotrophin, estrogen, MAPK, and PI3K-Akt pathway (see Supplementary Table S1 online).

## Discussion

Recently, the repurposing of existing drugs has been proposed as a strategy for new drug development^11,12^. In the current work, we selected a systematic computation framework to explore potential treatment options for PD based on existing data about diseases, drugs and drug targets.

Since drugs usually interact with specific targets to exert an effect on biological processes, and drug targets always interact with disease-related genes, we collected PD-associated genes. GO enrichment analysis of all genes showed that the most enriched terms were oxidative respiratory chain, energy metabolism and ion transport, which are consistent with prior findings^13–15^. These findings established the foundation for further mechanistic studies and provided novel targets for therapy. It is well recognized that PD, as a complex disease, may be caused by mutations of multiple genes or by the dysfunction of multiple biological processes. Earlier studies have shown that disease genes tend to interact in cellular networks^16^. We calculated a score to predict the proximity between the drug targets and PD-related genes by integrating the information in the PPI networks and kept the drugs with high proximity as candidates.

The major features of PD pathology are the loss of dopaminergic neurons from the midbrain and the presence of αSyn protein inclusions. Hence, we chose profiles of neural cell lines to perform the IGSEA procedure. Eventually, ten drugs were kept after filtration.

Among the ten candidates, six drugs have been approved by the Food and Drug Administration (FDA) for clinical use in cancer patients. For instance, carfilzomib and afatinib are epidermal growth factor receptor (EGFR) inhibitors. Interestingly, EGFR gene polymorphisms were reported to be related to the susceptibility to PD^17^. Vorinostat (also known as a histone deacetylase inhibitor, which is an effective anti‐neoplastic agent for different types of tumors) has recently been reported to be a potential novel candidate for treating PD^18^. However, there are no reports about the correlations of tucatinib, tazemetostat and avapritinib with PD pathology. Two of these candidates are hormones. To date, few effective treatments for PD have been reported, but a phase 1/2a clinical trial (Identifier: NCT04127578) is ongoing, the aim of which is to characterize the potential efficacy of methylprednisolone for treating patients with PD who have at least one GBA1 mutation, and we look forward to witnessing more promising discoveries. Isosorbide mononitrate (ISMN) is a candidate treatment for cerebral small vessel disease and lacunar ischemic stroke^19^. Arsenic trioxide showed beneficial effects on patients with acute promyelocytic leukemia^20^ and systemic lupus erythematosus (SLE) in a mouse model^21^. Inflammasomes might be involved in the therapeutic mechanism of arsenic trioxide in the above diseases. However, these two drugs have not yet been investigated in PD.

Nine differentially expressed drug-target genes with p < 0.05 were screened, and PSMB10, SLC47A2, HDAC8, and BCL2 were clustered into the same model, while KIT, TXNRD1, JUN, AKT1, and PML were clustered into another one, suggesting that these nine targets may act through distinct mechanisms. Sun et al.^22^ found that three beta subunits of immunoproteasome (PSMB9, PSMB10, PSMB8) all colocalized with α-syn, and PSMB9 knockdown aggravated the accumulation of α-syn in a cell model of PD. BCL2 overexpression protects dopaminergic neurons against neurodegeneration^23^ and it may play a role in dopaminergic development and PD^24^. Stéphane et al. showed that eliminating Jun N-terminal kinases (JNKs) can prevent neurodegeneration and improve motor function in an animal model of PD^25^. Another study suggested that activating the Akt1-CREB pathway might halt neurodegeneration in PD^26^. Furthermore, we found no experimental studies that have focused on PD in association with SLC47A2, HDAC8, TXNRD1, PML, and KIT, so this requires more observational data for verification.

Further KEGG pathway analysis revealed that these nine genes were enriched in the apoptosis, neurotrophin, estrogen, MAPK, and PI3K-Akt pathway. Some of them may represent novel targets for therapeutic intervention. Apoptosis is considered the main mechanism of neuronal death in PD, which could be targeted as possible therapies for PD^27^. In a randomized control trial (RCT), the effects of glial cell line-derived neurotrophic factor (GDNF) in Parkinson's disease were investigated^28^. MAPK consists of 3 subfamilies, ERK, JNK and P38. Results from the PD model implicate that selective inhibitors of p38 may help preserve the surviving neurons in PD and slow down the disease progression^29^.

In the last decade, multi-target drugs have attracted considerable interest in the treatment of complex diseases. The multi-target ligands have clear advantages, such as more predictive pharmacokinetics and reduced risk of drug interactions. For example, the multi-receptor approach for the Cannabinoid Receptor Subtype 2 was proposed for cancer and neurodegeneration therapy^30^. Bromophenols is a ligand for both dopaminergic receptors and human monoamine oxidase^31^. This study may provide new insights for revealing novel potential drugs and targets for multi-target drug screens. Additionally, the present strategy based on repositioning drugs could provide valuable clues for drug design and exploration for the treatment of other disorders.

However, there were also several limitations of this study. First, a potential drawback of proximity analysis is that it relies heavily on known information, including genes, drugs and targets, but this information is still far from being completely understood. Second, although some of the drugs extracted appear to be good candidates for further investigation, it is uncertain whether any of them would actually be effective for PD. More investigations are needed to determine the best use of these drugs to minimize side effects and to maximize patient benefit. Additionally, future studies should pay more attention to the novel targets, not just the drugs themselves.

In summary, this network-based approach enabled us to identify several novel drug candidates and targets that could been applied in treating PD. Although these results are still in the preliminary stages, they will provide clues for further experimental exploration. Additional investigation of these drugs and gene networks could lead to better preventive strategies for PD.

## Materials and methods

The workflow of this study is presented in Supplementary Fig. S2 online.

### DrugBank and expression data preprocessing

The drug–target relationships were constructed based on the DrugBank database^32^, which contained 13,680 drug entries and 4875 corresponding targets. The gene expression data were downloaded from Gene Expression Omnibus (GEO, http://www.ncbi.nlm.nih.gov/geo). The dataset with the largest sample size (GSE99039)^33^ was selected as the reference group. The platform data is GPL570 Affymetrix Human Genome U133 Plus 2.0 Array, and 233 healthy controls and 205 PD patients are included. After log2‐ transformation, the data were centered and scaled for differentially expressed genes (DEGs) analysis by limma Package^34^ of R software (version 3.6.4)^35^. DEGs were defined by a p-value < 0.05.

### Collection of PD-related genes

Genes associated with PD were retrieved from KEGG (https://www.genome.jp/kegg/)^36,37^, OMIM (https://www.ncbi.nlm.nih.gov/omim), Genotype (https://www.ncbi.nlm.nih.gov/gap/phegeni), DISEASES (https://diseases.jensenlab.org/Search), and HGMD (http://www.hgmd.cf.ac.uk/). This was done by querying the above databases using the “Parkinson’s disease” keyword. Genes from the above five databases were combined and mapped to their corresponding HUGO gene nomenclature committee^38^ (HGNC)-based official gene symbols. Duplicate genes and genes of unknown function were removed, and all remaining genes were retained as the PD-related genes.

### Protein–protein interactions between the PD-related genes

Proteins are the molecules that execute most cellular functions and many regulatory processes take place at this level, and biomolecules always achieve certain functions through extensive interactions with other proteins. To evaluate the correlations between the PD-related genes, we adopted the protein–protein interactions (PPI) network-based approach. The PPI data were obtained from the STRING^39^, PINA^40^ and HuRI databases. In the STRING database, the combined score is computed by combining the probabilities from the different evidence channels and corrected for the probability of randomly observing an interaction^41^. In this study, the combined score is calculated based on experiments, databases, co-expression, neighborhood, co-occurrence, and co-expression. STRING interactions with a combined score of 0.9 or higher were retained. All proteins retrieved from PINA and HuRI were preserved. After outlier removal, a PPI network was constructed based on the common interplayed relationships of three databases, and then visualized using the Cytoscape software (version 3.7.2)^42^.

### Enrichment analysis of the PD-related genes

Functional enrichment analysis is often conducted to investigate the potential mechanism of the gene set of interest. Gene Ontology (GO) annotation and KEGG analysis are the most commonly used methods. GO provides the classification of gene functions, the relationships between genes of interest in three categories (GO: biological process, GO: cellular component, and GO: molecular function). The KEGG analysis is applied to explore potential signaling pathways that genes may participate in^43^. The GO annotation and KEGG pathway enrichment analysis were performed using an R package “clusterProfiler”^44^. Only the terms/pathways with a false discovery rate (FDR) < 0.05 were considered significantly enriched in this work.

### Network-based proximity between drugs and PD

To uncover the targeting genes of drugs, we also drew upon the new approach from prior studies^45^ to calculate the distance between drugs and PD-related genes. Given *G*, the PD-related genes-set; *T*, the set of drug targets, the distance *d(g,t)*, namely the shortest path length between nodes *g* (*g*_∈_*G*) and *t* (*t*_∈_*T*) in the network, was calculated as below:

1 $$ d\left( {G,T} \right) = \frac{1}{{\left| T \right|}}\mathop \sum \limits_{{t \in T}} \min _{{g \in G}} \left( {d\left( {g,t} \right) + w} \right) $$

*w*, the weighted-score of a target; *w* = *− *ln(*D* + 1) if a target is in the PD-related genes-set; if not, *w* = 0. *D*, the PPI degree of PD-related genes.

The significance of relatedness between a drug and PD was evaluated using a reference distance distribution corresponding to the drug. Specifically, a set of proteins (*P*) matched to the number of drug targets was randomly selected in the network. The distance *d(G,P)* between these proteins and PD-related genes was computed. We repeated the randomization process 10,000 times and achieved the reference distribution. The mean *μ*_*d(G,P)*_ and standard deviation σ_*d(G,P)*_ of the reference distribution were used to calculate a z-score by converting observed distance to a normalized distance, i.e., proximity value:

2 $$ z\left( {G,T} \right) = \frac{{d\left( {G,T} \right) - \mu _{{d\left( {G,P} \right)}} }}{{\sigma _{{d\left( {G,P} \right)}} }} $$

### Calculation of drug signatures

The drug-perturbed gene expression profiles were derived from the Library of Integrated Network-based Cellular Signatures (LINCS)^46^, which is based on gene expression changes that describe the response of various types of cells when exposed to different agents. 165 nervous system-related datasets were obtained. Then, each filtered dataset was subject to Inverted Gene Set Enrichment Analysis (IGSEA) with the PD-related genes^45,47^, the enrichment score (ES), and the nominal p-value for quantifying enrichment magnitude and statistical significance of the genes. Finally, multiple comparisons among all the expression datasets were performed using the Benjamini–Hochberg FDR method. The gene set with FDR < 0.25 after performing 1,000 permutations was considered significantly enriched for gene expression datasets, and the corresponding drugs were deemed to be potential candidates for PD.

### Differentially expressed drug-target genes analysis

A gene that acts as both an effective drug target and a differentially expressed site could emerge as an important therapeutic target for the treatment. So, we took the intersection of DEGs and drug-target genes, i.e., differentially expressed drug-target genes, compared the expression distribution of these genes between the two groups of samples, and visualized with a box diagram.

## Supplementary Information

Supplementary Information.

## Data Availability

The gene expression data analyzed during the present study are available in the GEO with the accession number GSE99039.
