# Supplementary material for: Integrated network analysis identifying potential novel drug candidates and targets for Parkinson's disease
Source: Sci Rep. 2021 Jun 23;11:13154. doi: 10.1038/s41598-021-92701-2 (PMC8222400; doi:10.1038/s41598-021-92701-2)
Supplement: Supplementary file 1 — Supplementary Information. [file 41598_2021_92701_MOESM1_ESM.pdf]

Supplementary material

Integrated network analysis identifying potential novel drug candidates and targets for Parkinson's disease

Pusheng Quan, Kai Wang, Shi Yan, Shirong Wen, Chengqun Wei, Xinyu Zhang, Jingwei Cao, Lifen Yao*

Supplementary Figures

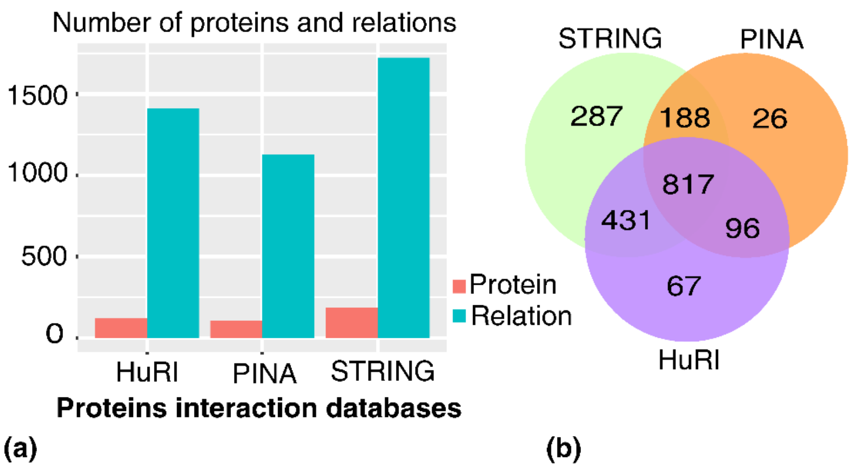

Supplementary Figure S1: PPI collection. (**a**) Bar chart showing the number of proteins and interactions obtained from three databases. (**b**) Veen diagram show the number of protein-protein interactions collected from three databases. PPI, protein-protein interactions.

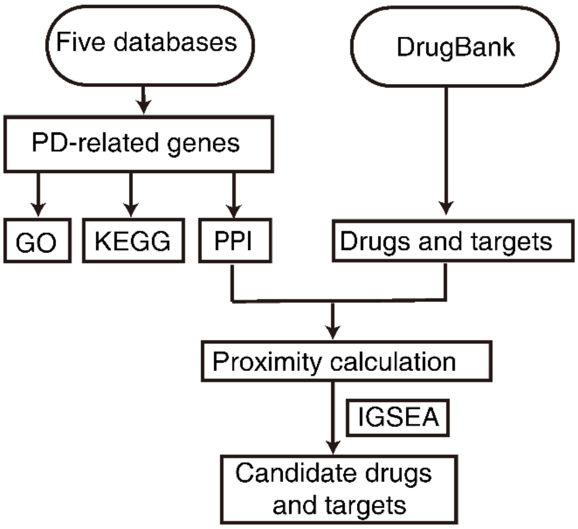

Supplementary Figure S2. The workflow of the study.

Supplementary Table

Supplementary Table S1. GO and KEGG enrichment analysis of the nine differentially expressed drug-target genes.

Category	Term	FDR	GC*	Gene ID
GO-BP	GO:0006979~response to oxidative stress	0.000314789	5	BCL2/TXNRD1/JUN/AKT1/PML
	GO:0009314~response to radiation	0.000314789	5	BCL2/KIT/JUN/AKT1/PML
KEGG	hsa04722: Neurotrophin signaling pathway	0.003836	3	BCL2/JUN/AKT1
	hsa04210: Apoptosis	0.003836	3	BCL2/JUN/AKT1
	hsa04915: Estrogen signaling pathway	0.003836	3	BCL2/JUN/AKT1
	hsa04010: MAPK signaling pathway	0.01463	3	KIT/JUN/AKT1
	hsa04151: PI3K-Akt signaling pathway	0.017499	3	BCL2/KIT/AKT1

GC*: Gene count
